# Yellow fever vaccine protects mice against Zika virus infection

**DOI:** 10.1371/journal.pntd.0009907

**Published:** 2021-11-04

**Authors:** Ana C. Vicente Santos, Francisca H. Guedes-da-Silva, Carlos H. Dumard, Vivian N. S. Ferreira, Igor P. S. da Costa, Ruana A. Machado, Fernanda G. Q. Barros-Aragão, Rômulo L. S. Neris, Júlio S. dos-Santos, Iranaia Assunção-Miranda, Claudia P. Figueiredo, André A. Dias, Andre M. O. Gomes, Herbert L. de Matos Guedes, Andrea C. Oliveira, Jerson L. Silva

**Affiliations:** 1 Laboratório de Biologia Estrutural de Vírus, Instituto de Bioquímica Médica Leopoldo de Meis, Universidade Federal do Rio de Janeiro, Rio de Janeiro, Brazil; 2 Instituto Nacional de Ciência e Tecnologia de Biologia Estrutural e Bioimagem, Centro Nacional de Biologia Estrutural e Bioimagem (CENABIO), Universidade Federal do Rio de Janeiro, Rio de Janeiro, Brazil; 3 Laboratório de Termodinâmica de Proteínas e Vírus Gregorio Weber, Instituto de Bioquímica Médica Leopoldo de Meis, Universidade Federal do Rio de Janeiro (UFRJ), Rio de Janeiro, Brazil; 4 Faculdade de Farmácia, Universidade Federal do Rio de Janeiro, Rio de Janeiro, Brazil; 5 Laboratório de Imunobiotecnologia, Instituto de Microbiologia Paulo de Góes, Centro de Ciências da Saúde, Universidade Federal do Rio de Janeiro, Rio de Janeiro, Brazil; 6 Laboratório de Imunofarmacologia, Instituto de Biofísica Carlos Chagas Filho, Universidade Federal do Rio de Janeiro, Rio de Janeiro, Brazil; 7 Laboratório de Microbiologia Celular, Instituto Oswaldo Cruz (FIOCRUZ), Rio de Janeiro, Brazil; Instituto Evandro Chagas, BRAZIL

## Abstract

Zika virus (ZIKV) emerged as an important infectious disease agent in Brazil in 2016. Infection usually leads to mild symptoms, but severe congenital neurological disorders and Guillain-Barré syndrome have been reported following ZIKV exposure. Creating an effective vaccine against ZIKV is a public health priority. We describe the protective effect of an already licensed attenuated yellow fever vaccine (YFV, 17DD) in type-I interferon receptor *knockout* mice (A129) and immunocompetent BALB/c and SV-129 (A129 background) mice infected with ZIKV. YFV vaccination provided protection against ZIKV, with decreased mortality in A129 mice, a reduction in the cerebral viral load in all mice, and weight loss prevention in BALB/c mice. The A129 mice that were challenged two and three weeks after the first dose of the vaccine were fully protected, whereas partial protection was observed five weeks after vaccination. In all cases, the YFV vaccine provoked a substantial decrease in the cerebral viral load. YFV immunization also prevented hippocampal synapse loss and microgliosis in ZIKV-infected mice. Our vaccine model is T cell-dependent, with AG129 mice being unable to tolerate immunization (vaccination is lethal in this mouse model), indicating the importance of IFN-γ in immunogenicity. To confirm the role of T cells, we immunized nude mice that we demonstrated to be very susceptible to infection. Immunization with YFV and challenge 7 days after booster did not protect nude mice in terms of weight loss and showed partial protection in the survival curve. When we evaluated the humoral response, the vaccine elicited significant antibody titers against ZIKV; however, it showed no neutralizing activity *in vitro* and *in vivo*. The data indicate that a cell-mediated response promotes protection against cerebral infection, which is crucial to vaccine protection, and it appears to not necessarily require a humoral response. This protective effect can also be attributed to innate factors, but more studies are needed to strengthen this hypothesis. Our findings open the way to using an available and inexpensive vaccine for large-scale immunization in the event of a ZIKV outbreak.

## Author summary

Zika virus (ZIKV) is as an important infectious that may result in severe congenital neurological disorders. Our study reports that the current attenuated yellow fever vaccine is effective in immunizing against the infection caused by the Zika virus, due to the similarity between the two viruses. To study the efficacy of the vaccine, we used different mouse strains, including both animals with a healthy immune system (immunocompetent) and animals with compromised immune systems and therefore more susceptible to viral (immunocompromised) infections. The vaccine was given subcutaneously, as it does in humans. The animals were inoculated with the Zika virus directly into the brain—a protocol normally adopted in vaccine studies to simulate a high lethality infection. In all cases, the vaccinated mice developed a high degree of protection against Zika infection. Altogether, we demonstrate that the YFV vaccine elicits an immune response that protects against cerebral infection by ZIKV. Our findings suggest the possibility of using an available and inexpensive vaccine for large-scale immunization in the event of a ZIKV outbreak.

## Introduction

Zika virus (ZIKV) probably emerged in the early 1900s and remained undetected for several years [1]. This virus was first isolated in 1947 from a sentinel rhesus monkey (*Macaca mulatta*) presenting with a febrile illness in the Zika Forest of Uganda [2]. The first case of ZIKV in humans was reported in 1952 [3], and ZIKV was historically regarded as a self-limiting disease. However, the scenario began to change in 2013, when a large outbreak in French Polynesia was associated with cases of Guillain-Barré syndrome [4]; during an outbreak in Brazil (2014–2015), authorities reported an increased number of children born with microcephaly [1,5]. ZIKV infection is known to be associated with congenital malformations and other neurological complications, such as Guillain-Barré syndrome [6,7]. Over the years, the epidemiological scenario of ZIKV has expanded quickly and has been considered endemic not only in Latin America but also in Caribbean regions and in parts of Africa and Asia [8].

Different vaccine models, including inactivated and attenuated models, have been tested in preclinical studies [1,9,10]. Some of these models have shown success in mice, and some of them have advanced to the clinical stage [1,9,10]. Infectious agents may lead to protection against other distinct but similar infectious agents [11]. This mechanism is known as cross-protection and was, for example, the basis of the first vaccine to be developed, which led to the global eradication of smallpox [12]. Members of the *Flaviviridae* family are similar, and some members of this family are the targets of currently available vaccines, such as the attenuated yellow fever virus (YFV) vaccine [13]. It was previously suggested that the low coverage of YFV vaccine, especially in the Northeast Region of Brazil, might be related to the high number of cases and microcephaly caused by Zika [7]. On the other hand, the number of Zika cases was apparently reduced under increased YFV vaccine coverage after an outbreak of yellow fever in the southwest. Based on the cross-protection observed for different vaccines, we hypothesized that this protection was induced by YFV against ZIKV infection. YFV and ZIKV are flavivirus and share several T cells epitopes that can work in cross-protection evoking mechanism associated to protection. A common T cell epitope activated during YFV vaccination, after Zika challenge, could rapidly be recruited and perform effector functions [14]. Furthermore, mechanisms of trained immunity, another type of non-specific cross-protection, can contribute to control the infection through epigenetic reprogramming [15]. Thus, it would be extremely interesting to use an already licensed vaccine with efficacy potential against ZIKV infection that could be deployed quickly in cases of ZIKV outbreaks.

Here, we evaluated whether a vaccine for YFV, a flavivirus that is similar to ZIKV, could prevent or at least decrease the severity of disease caused by ZIKV via a cross-protection mechanism and performed a follow-up of the survival, behavioral, and neuropathological consequences of infection. We used the attenuated YFV 17DD vaccine because it is a vaccine model that has long been used in humans with well-established tolerability. YFV vaccines have the advantage of already being licensed, and they can be safely used in humans. Despite the short-term protection observed, our results suggest a positive modulation against Zika infection promoted by YFV 17DD vaccination, raising the possibility of using this already commercialized vaccine.

## Results

### YFV vaccine is safe for use in A129 and BALB/c mice

**Fig 1:**
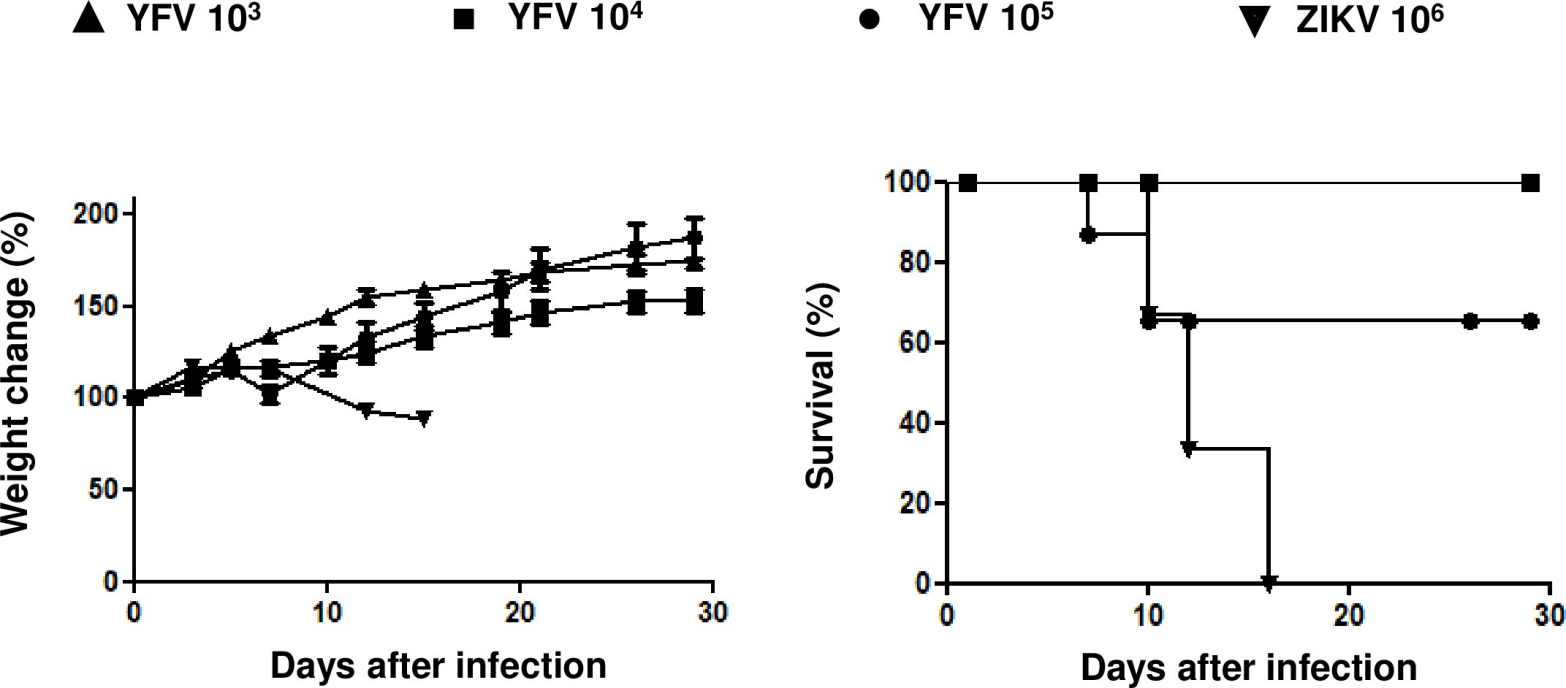
Dosing analysis of the YFV vaccine (subcutaneous route) in interferon-1 receptor knockout mice (A129). The mice were subcutaneously vaccinated with different doses of YFV 17DD (10^5^, 10^4^, or 10^3^ PFU) or challenged subcutaneously only with ZIKV (10^6^ PFU) as a control. The weights **(A)** and survival **(B)** were measured. N = 5; Statistical Analysis: For the weight change, we used one-way ANOVA, and no significant difference was observed. For survival, the log-rank (Mantel-Cox) test was used. *p*<0.01.

Based on a hypothesized cross-reaction between the YFV vaccine and ZIKV, we evaluated the tolerability of the attenuated YFV 17DD vaccine in A129 mice, monitoring both weight loss and mortality after two immunization doses of the YFV vaccine. We tested three different doses of the YFV vaccine, namely, 10^5^, 10^4^ and 10^3^ plaque-forming units (PFU), and in parallel, we carried out the challenge of mice only with a lethal 10^6^ PFU dose of ZIKV (S1 Fig) as a control group (Fig 1). In animals immunized with YFV, although there was no difference in the weight change (Fig 1A), we observed 35% death of mice at the 10^5^ YFV dose (Fig 1B), while the immunized animals with 10^4^ and 10^3^ PFU doses of YFV remained asymptomatic; in contrast, nonimmunized animals challenged only with ZIKV lost weight (Fig 1A) and died (Fig 1B). Thus, we chose a YFV dose of 10^4^ PFU, which had no apparent effects, and adopted this dose for subsequent experiments in mice.

### YFV vaccine induces protection against ZIKV infection in A129 mice

**Fig 2:**
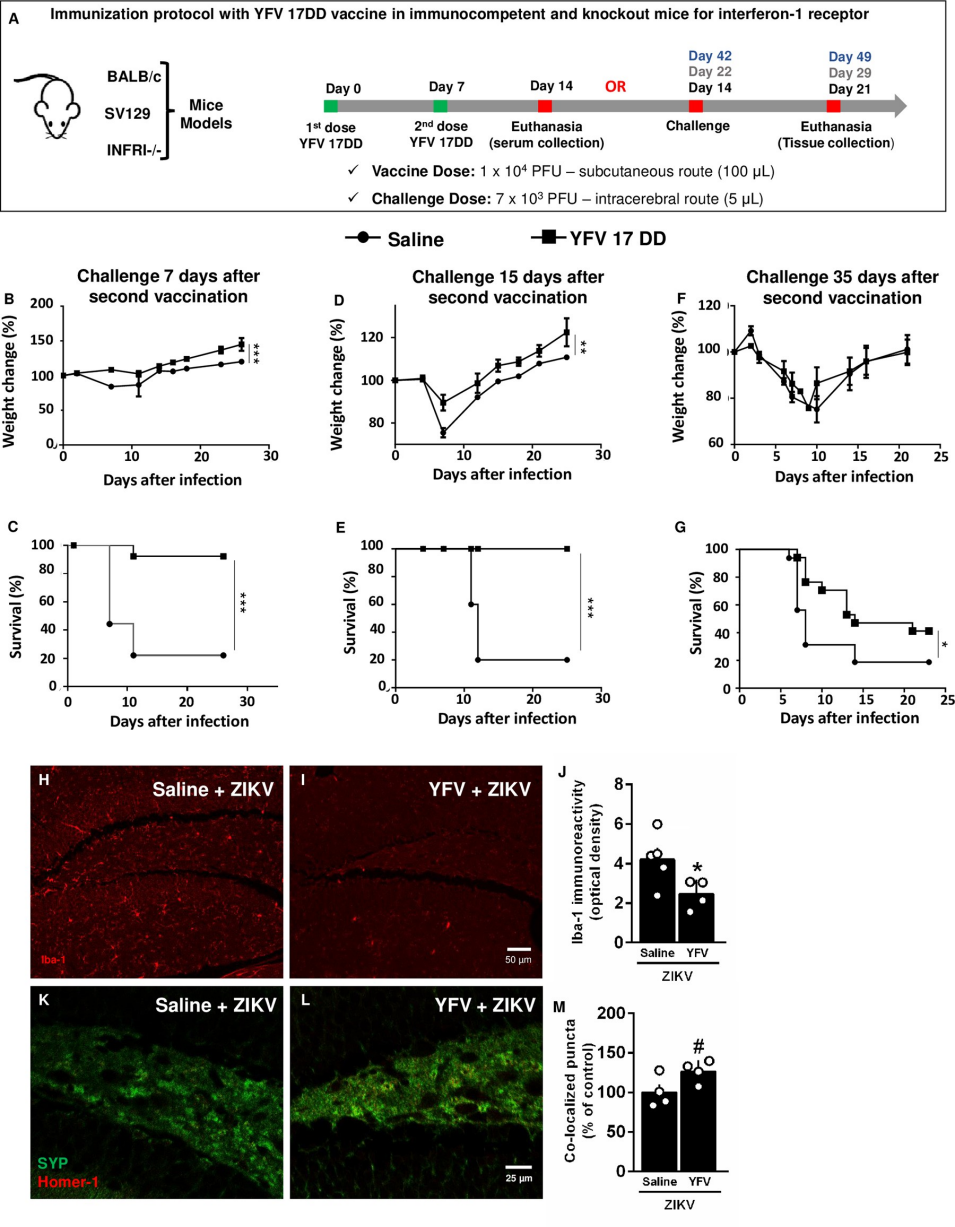
YFV vaccine protects interferon-1 receptor knockout mice (A129) against intracerebroventricular (icv) challenges with ZIKV. Immunization protocol with YFV 17DD in mice (**A**). The mice were challenged 7 days (**B** and **C**), 15 days (**D a**nd **E**) and 35 days (**F** and **G**) after infection, and their weight loss and mortality were monitored. Statistical Analysis: The Mantel-Cox test was used for the survival curves. Two-way ANOVA was used for the weight curves. N = 5. The 35-day experiment is represented by N = 16 from three different experiments. *p<0.05, **p<0.01, and ***p<0.0001. (H-M) Immunized animals were challenged with ZIKV by the intracerebroventricular route 15 days after the vaccine, and their brains were collected for morphological analysis after 7 days. (H-I) Representative images of Iba-1 (phagocytic cell marker) immunostaining in the hippocampus of mice. (J) Quantification of Iba-1 immunoreactivity in the hippocampus of mice. Bars represent mean ± SEM. Symbols represent individual mice. Unpaired t-test, *p < 0.05. (K-L) Representative images of synaptophysin presynaptic marker (SYP, green) and homer-1 postsynaptic marker (red) colocalized puncta (yellow) in the hippocampus of mice. (M) Synaptic puncta quantification in the hippocampus of animals. Bars represent mean ± SEM. Symbols represent individual mice. Unpaired t-test, #p < 0.075.

The susceptibility of the A129 strain to ZIKV infection was demonstrated previously [16] (Fig 1), making the A129 mouse a useful model to study ZIKV infection. The mortality in A129 mice from ZIKV infection declines with age [16], and we adopted a short vaccine protocol period to challenge mice at an age at which they are more susceptible. We immunized the A129 mice twice with a 10^4^ PFU dose of YFV vaccine, with seven days between doses. Following immunization, the mice were challenged with ZIKV (7x10^3^ viral particles) via the intracerebral route (IC) at different intervals after immunization (7, 15, and 35 days after the second dose of YFV vaccine) (Fig 2). The attenuated YFV vaccine was effective at protecting susceptible animals, especially 7 (Fig 2B and 2C) and 15 days (Fig 2D and 2E) after immunization. The vaccinated mouse group gained more weight (Fig 2B and 2D) and presented much lower mortality (Fig 2C and 2E) than the saline-treated mouse group. The difference in mortality was more evident than the difference in weight loss because many of the unvaccinated mice rapidly lost weight and died within 10 days. Some of the mice that died after the tenth day (Fig 2C) lost less weight. At 35 days following the second dose (Fig 2F and 2G), the protection decreased but was still present. No difference was observed in the weight losses, but the mortality was statistically lower.

To evaluate the protective effect of immunization with 10^4^ PFU of YFV 17DD against the microglial phenomenon in the central nervous system induced by infection with ZIKV (7x10^3^ viral particles), we performed an immunohistochemical assay in the brain tissue of A129 mice vaccinated and challenged intracranially with ZIKV. Therefore, we found that YFV 17DD prevented the ZIKV-induced increase in hippocampal Iba-1 immunoreactivity in mice (Fig 2H–2J). On the other hand, synapse loss is a common feature of different neurodegenerative conditions. Thus, to evaluate whether immunized mice present protection against synapse loss induced by ZIKV infection, we quantified the colocalization between synaptophysin (SYP, a presynaptic protein) and Homer-1 (a postsynaptic protein) immunoreactive puncta, a measure of functional synapses, in the hippocampus of mice. In Fig 2K–2M, we demonstrated that ZIKV-infected animals immunized with YFV presented an increased number of synaptic puncta compared with nonimmunized mice (Fig 2K–2M). Altogether, these findings suggest that YFV protects mice against brain damage induced by ZIKV infection.

### YFV decreases viral load in ZIKV-infected SV129 mice

To confirm the protection of the YFV 17DD vaccine in an immunocompetent animal model, we evaluated the effects of a ZIKV challenge in SV129 mice (background of immunocompromised A129 animals) 35 days after vaccination. ZIKV viral loads were markedly lower, indicating that vaccination promotes a response against viral spread in the brain in wild-type mice (S2 Fig).

### YFV vaccine induces protection against ZIKV infection in BALB/c mice

**Fig 3:**
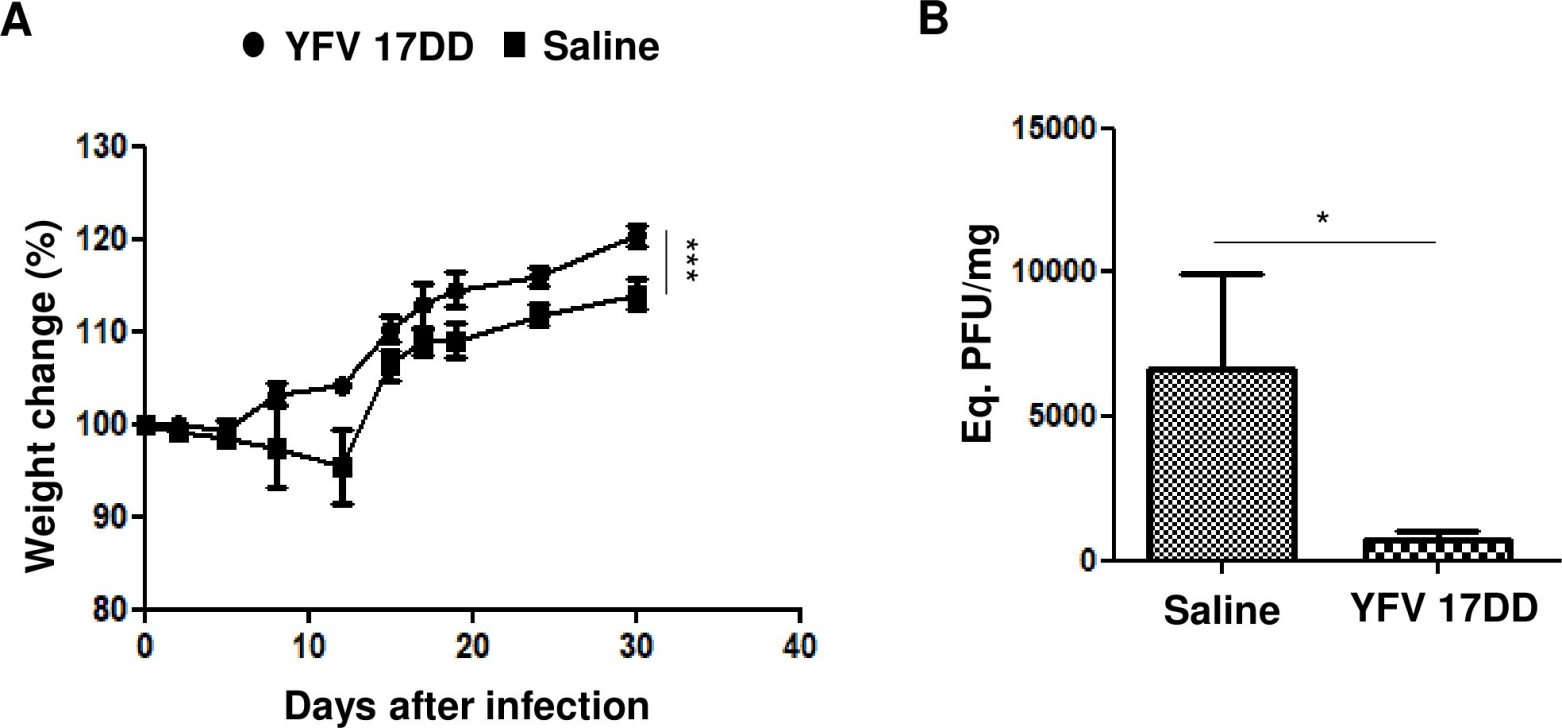
YFV vaccine protects immunocompetent BALB/c mice. The mice were challenged via the intracerebral route with 7x10^3^ ZIKV particles. Their weights were measured **(A),** and cerebral tissue qRT-PCR was performed 7 days after infection and ZIKV (PFU/mg are shown) **(B)**. N = 5 per group; statistical analysis: Changes in weight were analyzed by two-way ANOVA, and the qRT-PCR results were analyzed by the Mann-Whitney test. ***p<0.0001, *p<0.05.

We also tested the YFV vaccine in immunocompetent BALB/c mice. These BALB/c mice were immunized twice, and after 7 days, they were IC-challenged with ZIKV (following the same protocol used for the A129 mice described in Fig 2A). We observed that the vaccinated group presented no weight loss, while the saline group did (Fig 3A). The cerebral viral load was significantly different between the groups (Fig 3B), indicating that the prevention of clinical signs was correlated with lower viral propagation in the vaccinated mice.

### YFV vaccine protects BALB/c mice against neurological signs

We observed different neurological disturbances, such as spinning when suspended by the tail, shaking, hunched posture, ruffled fur and paralysis, following ZIKV infections in the BALB/c mice. We evaluated these manifestations in the vaccinated and saline groups after the challenge. All extremely recognizable clinical neurological signs were present in the saline group and completely absent in the vaccinated group (Table 1).

**Table 1:** Neurological signs in immunocompetent BALB/c mice after infection.

	Spin through tail suspension	Shaking, curved body and ruffled hair	Paralysis
**Saline group (N = 5)**	5/5 (100%)	5/5 (100%)	3/5 * (60%)
**Vaccine group (N = 5)**	0/5 (0%)	0/5 (0%)	0/5 (0%)

The mice were evaluated for the presence or absence of neurological signs by two independent observers. The signs were evaluated daily from the first day after infection.

* This animal recovered paw movement on the right side 15 days post infection.

In the saline group, 3 of the 5 animals presented an unsteady gait, which was marked by paralysis in at least one of the segments. In the vaccine group, no animals presented with this clinical sign. In 2 of the 3 symptomatic mice, unsteady gait was established as a permanent sequela (observed from 5 days after infection onwards). All the mice in the saline group exhibited agitation and touch sensitivity, but all the animals recovered from these behaviors. In the saline group, 3 animals showed spinning behavior during tail suspension. In 2 of these 3 animals, this behavior remained a sequela (which were observed from 5 days after infection onwards). In the vaccine group, no mice exhibited this behavior. These results indicate that the protective mechanism is efficient at controlling viral replication and brain damage, guaranteeing physiological homeostasis.

### YFV 17DD vaccine killed AG129 mice

We immunized AG129 mice (S3 Fig), in which both type 1 and type 2 interferon receptors are knocked out. We tested the 10^4^ and 10^2^ PFU doses at which the A129 mice were asymptomatic. The AG129 mice were highly susceptible to the YFV vaccine (all mice died after vaccination). Unfortunately, we were not able to evaluate the YFV vaccine on AG129 mice against Zika infection; however, this result suggests that IFN-γ is necessary to control YFV, which is absent on AG129 and could be necessary to confer cross-protection against a ZIKV challenge after YFV vaccination in A129 mice.

### YFV 17DD vaccine did not induce protection in nude (NU/J) mice against ZIKV infection

**Fig 4:**
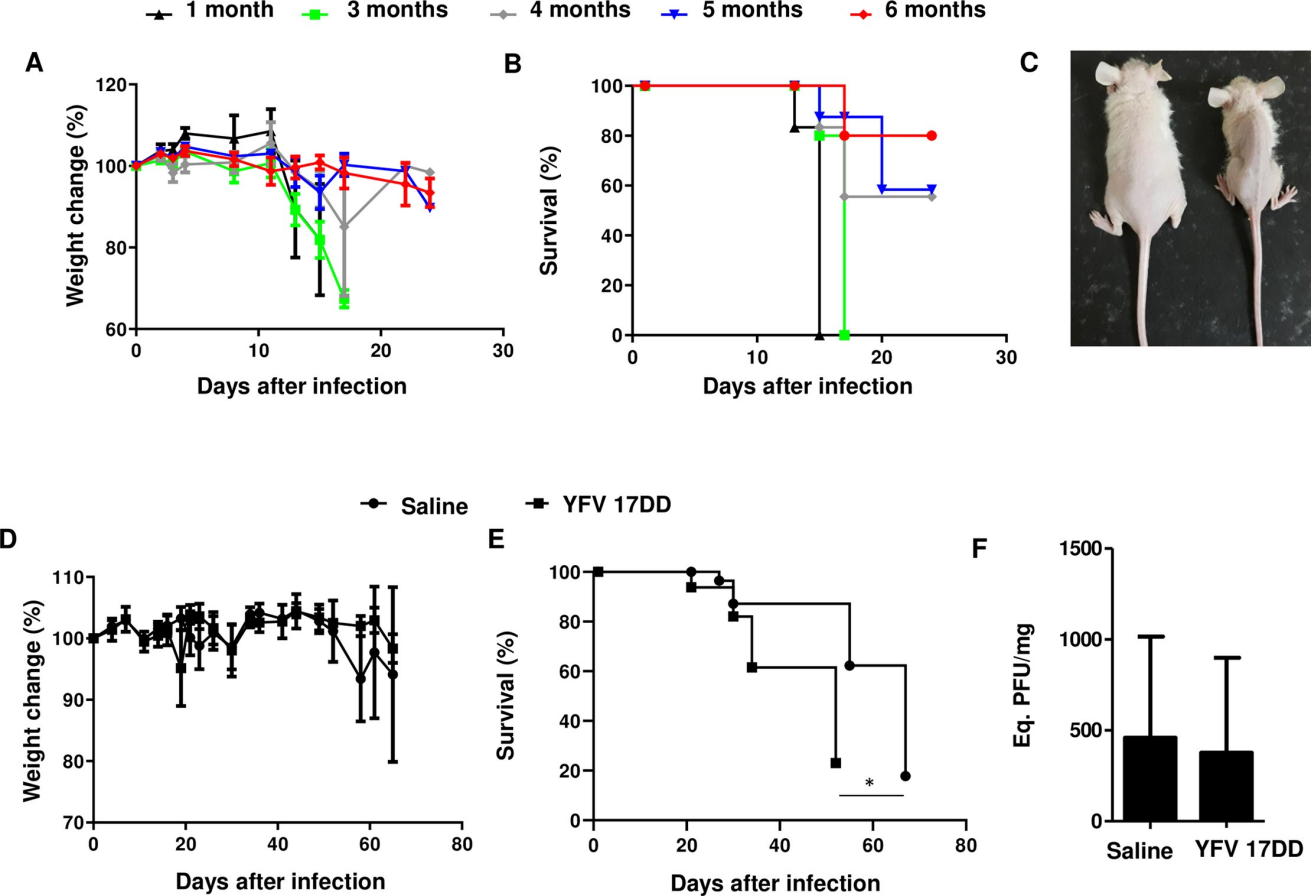
Nude mice are susceptible to infection, and YFV vaccination is ineffective. **(A)** Weight loss in nude mice at different ages after IC infection with 10^4^ ZIKV particles (N = 3 to 4 per group). **(B)** Survival curve for nude mice of different ages infected by the IC route (N = 3 to 4 per group). **(C)** Clinical aspects of mice infected with ZIKV (right animal) compared with noninfected mice (left animal). The nude mice were challenged with ZIKV after YFV 17DD vaccination to evaluate the dependence on the T cell response against ZIKV infection. **(D)** Weight change. **(E)** Survival curve for nude mice. **(F)** qRT-PCR. N = 7 per group. Statistical analysis: Mantel-Cox test for the survival curves. One-way ANOVA for the weight curves. *p<0.05.

Because of the importance of T cells in producing IFN-γ, we evaluated YFV 17DD in nude (NU/J) mice, which are deficient in T cells. We first analyzed ZIKV pathogenicity through the IC infection of mice of different ages (1, 3, 4, 5 and 6 months) (Fig 4A and 4B), demonstrating a relation with the immaturity of the immune system, as the mice are very susceptible at 1 and 3 months and partially susceptible at 4, 5 and 6 months, a phenotype that is very similar to that observed in A129 mice. We vaccinated the nude mice and then challenged them. No protection against death was detected (Fig 4E), but a delay in the survival curve was observed. In addition, vaccination did not promote any decrease in viral replication in the cerebral tissue (Fig 4F), which indicates the importance of T cell-mediated immunity for protection.

### Vaccination elicits nonneutralizing antibody production

**Fig 5:**
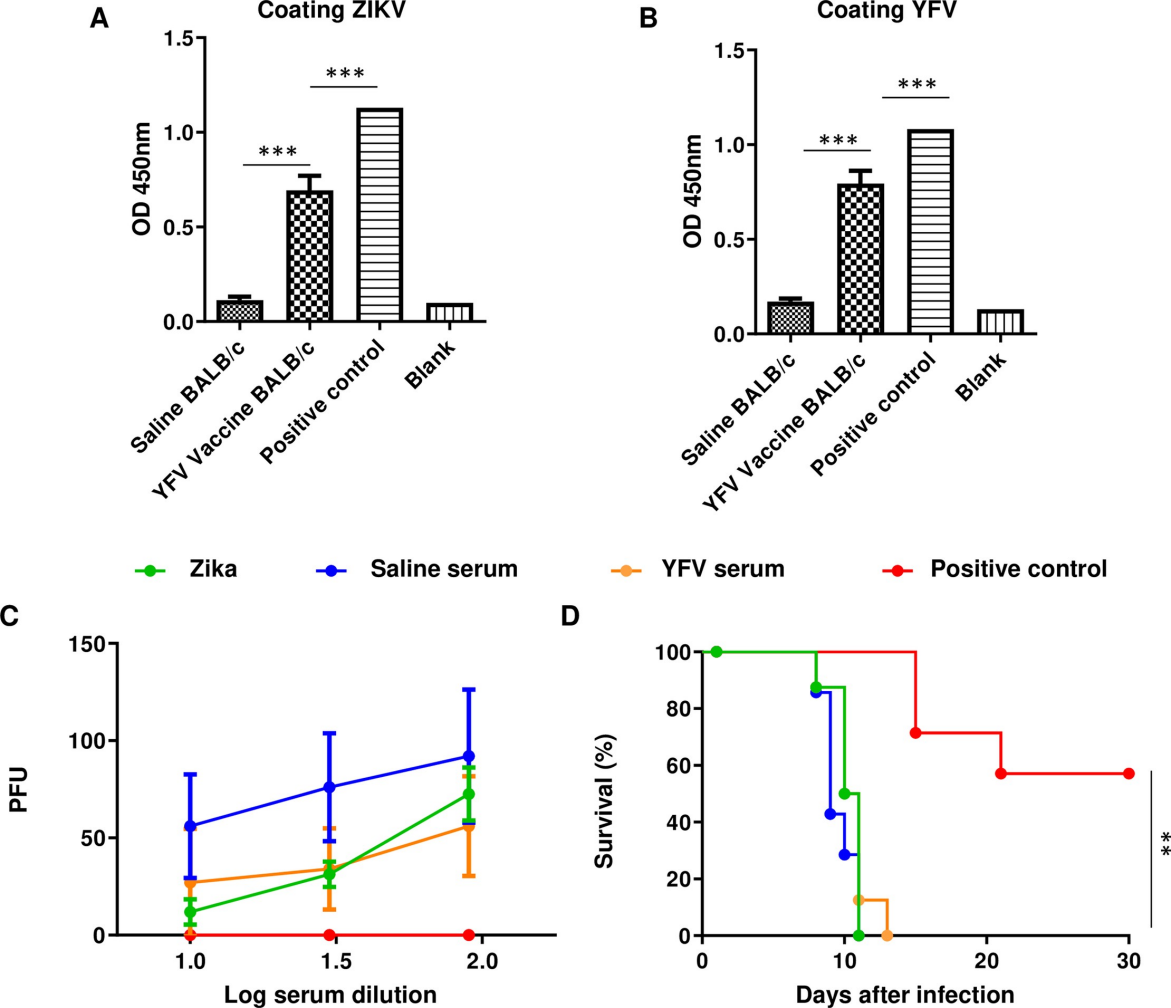
YFV vaccine elicits a specific IgG response in immunocompetent BALB/c mice. After the seventh day of the second vaccine dose, serum samples were collected and used to analyze the antibody response at a 1:360 dilution. **(A)** Antibody response by ELISA using ZIKV coating. **(B)** Antibody response by ELISA using viral YFV coating. All the serum samples differed from the saline group *** *p*≤0.0001. One-way ANOVA and Tukey posttest. **(C)** The sera were analyzed by testing their capacity to block ZIKV infections according to a microneutralization assay. PFU, plaque-forming units (greater PFU indicates less capacity to block infection). **(D)** The AG129 mice were challenged with a mixture of 10^4^ ZIKV particles with 4-fold diluted serum. A positive control was obtained by infecting mice 4 consecutive times (separated by 10 days each) with 10^4^ ZIKV particles by the intraperitoneal (IP) and intravenous routes, and samples were collected 10 days after the fourth infection. For microneutralization, all the groups differed from the positive control. **p≤0.01. Log rank (Mantel-Cox) test. For both ELISA and *in vitro* microneutralization, N = 10. *In vivo* microneutralization N = 8.

We also evaluated the ability of the antibodies produced against YFV to cross-react with ZIKV. We observed that immunizing the BALB/c mice induced the production of specific IgG antibodies against heterologous (ZIKV) and homologous (YFV) antigens (Fig 5A and 5B), which could be detected 7 days after booster immunization. This result indicated that the heterologous agent used in the vaccine (YFV) could elicit the production of antibodies that bind to ZIKV. We also evaluated the capacity of the antibodies produced against YFV to neutralize ZIKV infection in Vero cells. Our results demonstrated that the serum from the vaccinated mice did not neutralize the ZIKV infection (Fig 5C), whereas the serum from the mice infected with ZIKV did, suggesting that the mechanisms induced by YFV could not be related to the humoral immune response.

We also evaluated the possible protective effects of antibodies produced by vaccinated animals *in vivo*. AG129 mice have deactivated type 1 and type 2 interferon receptors and are highly susceptible to ZIKV infection. The application of the serum mixture of mice immunized with ZIKV was unable to protect these mice from infection (Fig 5D).

### Breastfeeding by immunized females is unable to protect infected pups from developing brain disorders

**Fig 6:**
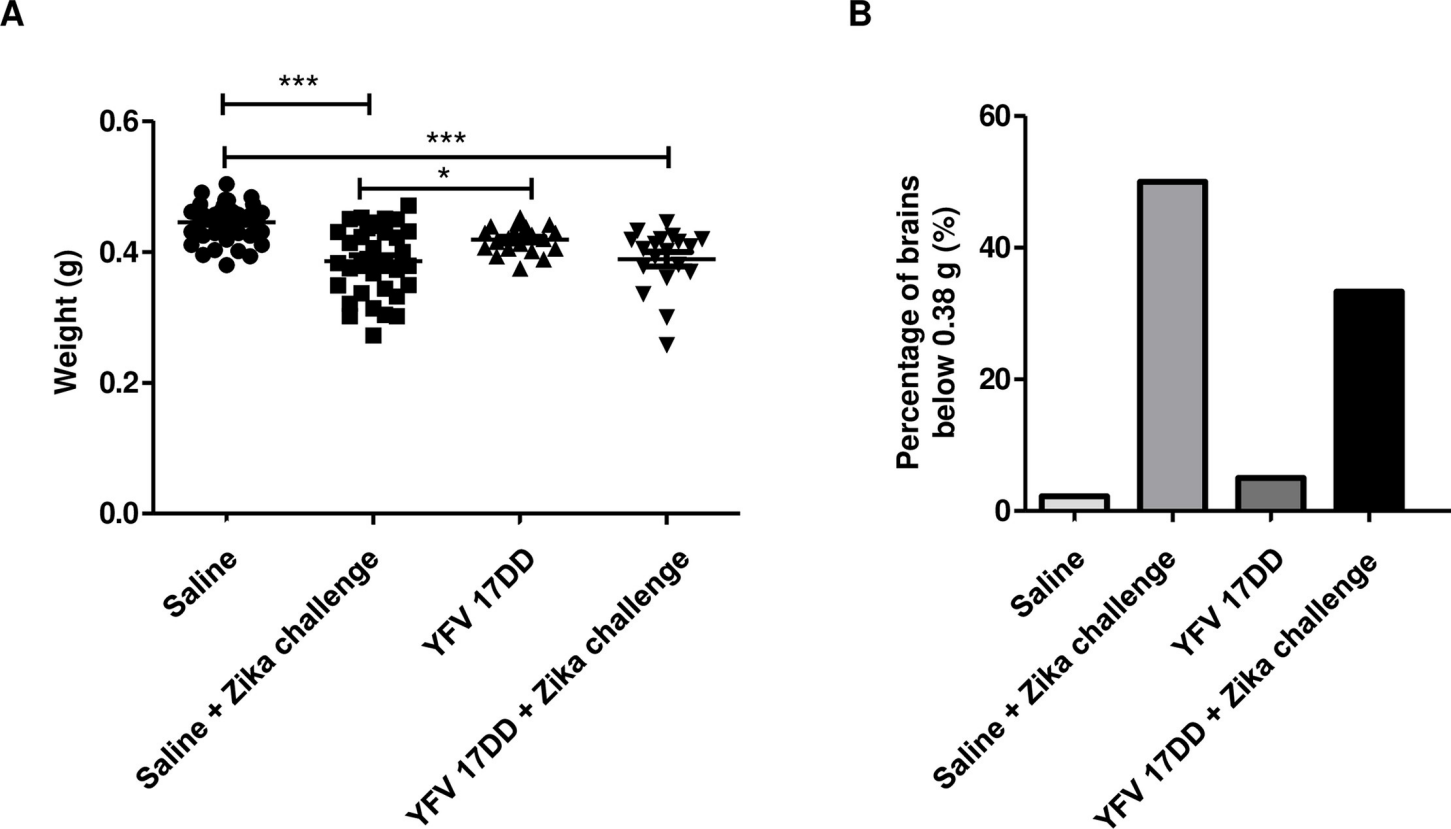
Effect of breastfeeding on pup brain development. After the seventh day, the female mice received the second dose of YFV 17DD vaccine or saline solution and were placed to mate. After birth, the mice were challenged subcutaneously with or without 10^6^ ZIKV particles. At 35 days after birth, the pups were euthanized, and the brains were removed and weighed. **(A)** Represents all groups with individual values. **(B)** The control mouse with the lowest brain weight in the saline group was selected as a cutoff point. The proportion of mice with lighter brains in the other groups was determined using this cutoff point. Statistics: One-way ANOVA with Tukey’s posttest. *p<0.05; ***p<0.01.

Female Swiss mice were divided into two groups: vaccinated and control. The first group received two doses of 10^6^ YVF 17DD, and the second group received two doses of saline. Seven days after the second dose, the mice were placed for crossing. Pregnant mice were separated into four groups: saline without challenge, saline + ZIKV challenge, vaccine without challenge and vaccine + ZIKV challenge. Three days after birth, Swiss mouse pups were subcutaneously challenged with 10^6^ ZIKV. After 35 days, the animals were euthanized, and their brains were weighed (Fig 6A). The infected animals had smaller brain sizes, and vaccination was unable to prevent the manifestation of this phenotype. The lightest brain in the saline group without challenge weighed 0.38 g, and we used it as a cutoff point. Then, we compared the proportion of brains in all the groups that were lighter than this cutoff point (Fig 6B). We observed that both infected groups had a higher proportion of lighter brains, with the vaccinated group showing a low proportion. This result supports the idea that YFV vaccination is effective at protecting adult mice but has low efficacy in promoting protection in pups through breastfeeding.

## Discussion

Vaccines against ZIKV have been studied following the outbreak in 2015. Different approaches, including using a virus inactivated by formalin and subunits or DNA vaccines, have been tested [1,9,10,17]. In this study, we immunized mice with the attenuated YFV 17DD vaccine and IC-challenged them with ZIKV infection. An IC infection in which ZIKV is inoculated directly into the CNS has a highly neurovirulent and pathogenic route; this route is considered a severe model of infection [18] and may require a strong immune response, which can probably be achieved only by using live vaccines, to protect the brain. YFV is one of the strongest immunogens ever developed because it confers long-lasting protection with a single dose [13].

In our first step, we standardized the YFV dose in A129 mice using immunization via the subcutaneous (SC) route. Recently, a similar result was observed by another group using a chimeric attenuated vaccine (ChimeriVax-Zika) based on YFV with ZIKV epitopes (premembrane and envelope genes from YFV were replaced by those from ZIKV) [19], which demonstrated that a dose of 10^5^ PFU resulted in a low mortality rate. This result is not surprising because attenuated vaccines, despite being safe, require some precautions for their use. When the tolerability of YFV (17-D) and ChimeriVax-Zika (CYZ) was analyzed in mice, CYZ was safer because it induced few deaths [19]. However, the study comparing the two vaccines involved the injection of 5-day-old mice via the IC route to evaluate their tolerability. Although this evaluation method is important, it does not reflect real conditions because vaccination does not occur via this route and is not performed in neonates. However, the YFV vaccine is recommended for people aged 9 months or older and has been used in pregnant women without any apparent adverse effects on the fetuses.

The A129 model has already been characterized for presenting, with wild-type YFV infection, viscerotropic disease and fatality after subcutaneous inoculation, despite the use of an attenuated vaccine strain [20]. Vaccination with 10^4^ PFU of YFV was shown to induce protection in A129 mice. The Giel-Moloney study that used ChimeriVax-Zika showed a reduction in the viral load in vaccinated A129 mice; however, no survival results were reported [19]. The protective efficacy of a live attenuated ZIKV vaccine with mutations in the NS1 gene and the 3’UTR of the ZIKV genome was evaluated only in pregnant mice, which did not allow us to compare those study results with our results [21]. In our work, YFV provided protection to immunocompromised mice infected by the IC route, and this protection was demonstrated by a reduction in the viral load in the brain and by increased survival but was time dependent. For SV129 mice, vaccination proved to be effective in controlling viral propagation in cerebral tissue, even with a challenge that occurred 35 days after the second vaccine dose (S2 Fig).

We also evaluated immunocompetent BALB/c mice. Recently, BALB/c mice were found to die after IC infection using 10^3^ or 10^4^ PFU, and some mice infected with 10^*2*^ PFU of ZIKV strain MR766 also died (Uganda, 1947) [22]. Other immunocompetent mice also died when infected at the neonatal stage, such as Swiss mice [23]. We observed that BALB/c mice did not die after an IC challenge with ZIKV, and our model allowed us to study neurological disorders as represented by easily recognizable clinical signs. Vaccination prevented the BALB/c mice from developing neurological disorders. Vaccination efficiently blocked viral propagation, which positively correlated with the clinical signs found in the BALB/c mice. Protection against an IC challenge requires a potent immune response not only because this route causes more severe disease but also because the CNS presents a level of isolation from the rest of the body (as an immune privileged site).

The mechanism of YFV vaccination that protects against YFV infection also involves neutralizing antibodies [24]. CYZ has been shown to elicit antibodies in mice and to reduce the viral load in a vaccinated group [19]. We detected antibody production against ZIKV (Fig 5A), but these antibodies did not have the capacity to neutralize ZIKV infection in Vero cells (Fig 5C). This finding has also been observed in AG129 mice, which are highly susceptible to infection. The mixture of the serum and the virus was not able to mitigate the infection (Fig 5D). The lack of protection in challenged pups whose mothers were previously vaccinated also supports this idea (Fig 6A and 6B). A study using a live-attenuated ZIKV vaccine showed protection through breastfeeding by antibodies present in milk [21]. In our study, if some significant amount of ZIKV neutralizing antibodies were present in the milk of the vaccinated mice, some level of protection would be expected. We observed that the YFV vaccine did not prevent microcephaly with breastfeeding in ZIKV-challenged pups.

As described above, the mechanisms of protection may not be dependent on the neutralizing activity of the antibodies. The protection observed 35 days post booster in A129 and Sv129 indicated short-term memory protection. To investigate this issue, we started with AG129 mice (S3 Fig). In our prior experiment, we observed that A129 mice are protected by YFV vaccination despite being sensitive to high doses of YFV. However, when we tested the 10^4^ or 10^2^ doses in AG129 mice (in which the A129 mice were asymptomatic), the animals were highly sensitive to YFV. At this dose, all the mice died. The induction of IFN-γ by YFV was demonstrated previously [25]. The YFV-17D vaccine induces a robust cellular immune response through the activation of a mixed Th1 and Th2 response, cytotoxic CD8+ T cells and a neutralizing antibody response [26]. These mixed responses are elicited by the activation of Toll-like receptors (TLRs), such as TLR2, TLR3, TLR7, TLR8 and TLR9, on dendritic cells [27]. Several CD4+ and CD8+ T cell epitopes have been characterized and are related to the protection induced by YFV vaccines [28,29], which suggests that future studies should assess possible cross-T cell epitopes between YFV and Zika.

The relative importance of NK and CD8+ cells in controlling early infection is known to vary between mouse strains, with T cells being more important in BALB/c mice [4]. Furthermore, the lack of this response in nude mice was positively correlated with the lack of protection. Although vaccination appeared to cause some delay in mouse death (Fig 4E), the total mortality in both groups was similar. This discrete protection effect may be attributed to innate factors. We cannot rule out a potential role of the innate immune system, as cross-protection induced by BCG vaccination has been observed. This mechanism has been linked to heterologous effects of adaptive immunity but also to potentiation of innate immunity through epigenetic mechanisms [30]. As a BCG vaccine, the YFV vaccine is an attenuated model, so in both cases, similar protection mechanisms may be elicited. Thus, the protection observed by YFV vaccination may partially involve trained immunity. YFV-17DD vaccination has been shown to comprise a complex network of cytokines in the innate immune compartment involving cytokines such as IFN-γ produced by NK cells [24]. Against Zika infection, YFV could induce protection using a combination of mechanisms involving adaptive immunity and trained immunity. The decreased protection observed with challenge occurring 35 days after vaccination is an indication that the protection observed is at least partially dependent on innate immunity.

Although we have shown that the protection observed against ZIKV by YFV 17DD vaccination does not come from the production of neutralizing antibodies, our study did not demonstrate the mechanistic part of cross-protection, but we hypothesized the role of the T cell response or even trained immunity in this protection observed. Further studies to provide the importance of T cells using CD4^-/-^ and CD8^-/-^ mice; and to investigate trained immunity using NLRP3^-/-^, CASP1/11^-/-^ and also to investigate the epigenetic signature in innate cells should be addressed to better understand this cross-protection mechanism.

Many ZIKV vaccine candidates are in the preclinical phase, and some are in clinical phases I and II. Different technologies, such as live attenuated vaccines, recombinant vector vaccines, subunit vaccines, whole inactivated vaccines, mRNA vaccines and DNA vaccines, have been tested [1,9,10,17]. Undoubtedly, the study and development of new vaccines is extremely important because these processes allow us to have the opportunity to test and develop more efficient and safer models. Some of these vaccine models may turn out to be highly effective, and some may not, but it will still take time to make these vaccines available. This time gap can be filled by the YFV vaccine, which has been used successfully for decades in the human population and is currently readily available. It is possible that the YFV vaccine may be effective at protecting humans against ZIKV, especially against neurological diseases in adults. The possibility of cross protection between flaviviruses is hypothesized. A recent epidemiological study reported that preexisting infection with dengue virus (as determined by high antibody titers) was associated with a reduced risk of ZIKV infection [31]. However, no experimental evidence has been provided for this hypothesis.

Concerning epidemiological data on the YFV vaccination of mothers of CZS infants, there are no systematic studies. However, a descriptive study indicated that Northeast Brazil had the lowest YFV vaccination coverage and was the region with the highest incidence of CZS between October 2015 and March 2016 [7]. If YFV truly protects against ZIKV in humans, it could provide a safe, quick and inexpensive vaccination model because its pros and cons in clinical practice are already well known. In addition, the YFV vaccine would be capable of protecting against two distinct pathogens simultaneously. Substantial time and resource savings could be accrued by using an already licensed vaccine. We believe that more studies on cross protection between flaviviruses are needed and that the use of the YFV vaccine during an outbreak of Zika may be strategic until a specific Zika vaccine is available.

## Materials and methods

### Ethics statement

All animal use involved in this work was approved by the Ethics Committee on the Use of Animals (CEUA) in Scientific Experimentation of the Health Sciences Center of the Federal University of Rio de Janeiro registered with the National Council for the Control of Animal Experimentation (CONCEA) based on Brazil regulations on the case number 01200.001568/2013-87.

### Cells

Vero (African green monkey kidney) cells (CCL 81) were obtained from the American Type Culture Collection (ATCC), Manassas, VA, USA, and cultured in high-glucose Dulbecco’s modified Eagle’s medium (Gibco DMEM; Thermo Fisher Scientific—Manassas, VA, USA). The culture medium was supplemented with 10% fetal bovine serum (FBS; Vitrocell Embriolife, Campinas, SP, Brazil) and 100 μg/mL streptomycin, and the cells were maintained at 37°C in a 5% CO_2_ atmosphere.

### Mice

We used different mouse strains in this study, namely, the immunocompetent BALB/c and SV129 strains and the immunocompromised A129 strain (IFNAR1), AG129 (IFNα/β/γR-/-) and nude (NU/J). In all experiments, four- to five-week-old mice were used before vaccination. All animals were obtained from UFRJ Central Biotherm (Rio de Janeiro/RJ, Brazil). All procedures were performed in accordance with the guidelines established by the Ethics Committee for Animal Use of UFRJ (CEUA 131/19).

### ZIKV and YFV

The ZIKV strain used in this study was ZIKVPE_243_ (GenBank ref. number KX197192), which was isolated from a febrile case in the state of Pernambuco, Brazil and was kindly given to us by Dr. Ernesto T.A. Marques Jr. (Centro de Pesquisas Aggeu Magalhães, FIOCRUZ, PE, Brazil). The YFV was YFV 17DD, which was kindly given to us by LATEV, Bio-Manguinhos/Fundação Oswaldo Cruz (Rio de Janeiro/RJ, Brazil). The viruses were propagated as described previously [23,32], and the viral titers were determined in Vero cells using a standard plaque assay at day 5 postinfection by crystal violet staining (Merck Millipore). The viral titers were determined in aliquots of harvested medium, and stocks of the viruses were stored at -80°C.

### Safety study

For the safety study, we injected the YFV vaccine at 10^3^, 10^4^ and 10^5^ PFU doses via the SC route into A129 mice, and we challenged the mice only with ZIKV 10^6^ PFU as a control.

### Vaccination and challenge

We performed two immunizations with attenuated YFV by the SC route using a dose of 10^4^ PFU with 7-day intervals between the doses. The mice were challenged with ZIKV by inoculating 5 μL of ZIKV (7x10^3^ viral particles) via the IC route using a 0.5 mL Hamilton syringe and 27 G ¼ needles. The control mice were treated with phosphate-buffered saline (PBS) instead of YFV. The challenged mice were observed for 4 weeks to evaluate their clinical signs, including ruffled fur, vocalization, shaking, hunched posture, spinning during tail suspension, paralysis and death. Dying animals were euthanized humanely. The protocol is summarized in Fig 2. Under an alternative protocol, the mice received only one dose, and seven days later, they were challenged.

### Assessment of neurological signs

After immunization with YFV 17DD and IC challenge with ZIKV (the protocol is summarized in Fig 2), the BALB/c mice were observed daily and analyzed for 60 min for clinical signs of infection by comparing the vaccinated infected and control groups with healthy mice. The animals underwent tail suspension for a maximum of 60 seconds to evaluate their neurological alterations. For this examination, the animals were tested twice daily with a minimum interval of 5 min between analyses.

### Immunohistochemistry

Mice were deeply anesthetized with xylazine (10 mg/kg, i.p.) and ketamine (100 mg/kg, i.p.) and perfused transcardially with PBS 0.1 M, pH 7.4, 50 mL per animal followed by ice-cold 4% paraformaldehyde. Brains were removed, postfixed for 24 h in the same solution, processed and embedded in paraffin. Slides containing coronal brain sections (5–8 μm) were subjected to antigen recovery by treatment with 0.01 M citrate buffer for 40 min at 95–98°C. Slides with mouse hippocampal tissue were incubated overnight with primary antibodies (rabbit anti-Iba1 1:500, WAKO #019–1941, mouse anti-synaptophysin 1:200, Vector Laboratories #S285; rabbit anti-Homer-1 1:100, Abcam #184955) diluted in PBS containing 3% BSA. Then, sections were incubated with Alexa 594- or 488-conjugated secondary antibodies (1,750; Invitrogen) for 1 h at room temperature, washed in PBS, and mounted in Prolong Gold Antifade with DAPI (Invitrogen). Synaptic puncta and microglial immunolabeling were imaged on a confocal microscope (Nikon) at ×630 magnification. Independent images of the hippocampus were used for analyses. For Iba-1 quantification, the total pixel intensity was defined for each image, and the data are expressed as integrated optical density (DO). In synaptic puncta analysis, each image obtained was a z-stack of 12–16 (0.33 μm depth) sections. We then used the Puncta Analyzer plugin in ImageJ 1.29 (NIH; RRID: SCR_003070) to count the number of colocalized, pre- (synaptophysin), or postsynaptic (Homer-1) puncta.

### Viral load determinations by qRT-PCR

Seven days post immunization (booster) with the attenuated YFV vaccine, the animals were challenged by the IC route. The viral load was measured in the brain tissue of the mice at day 7 post challenge (peak viremia in these models) by qRT-PCR using primers/probes specific for the ZIKV E gene as previously described [23]. The cycle threshold (Ct) values were used to calculate the log PFU/mg tissue equivalence after conversion using a standard curve with serial 10-fold dilutions of a ZIKV stock sample.

### Enzyme-linked immunosorbent assay (ELISA) evaluation of anti-mouse IgG levels in the serum of immunized immunocompetent mice

Polystyrene microplates (Corning, New York, NY, EUA) were coated overnight at 4°C with 10^5^ ZIKV or YFV viral particles. Following blocking for 2 h with PBS containing 1% bovine serum albumin (BSA) (LGC Biotecnologia, Cotia, SP), the serum from mice that were vaccinated with YFV was adsorbed in the wells at different concentrations and incubated overnight at 4°C. Then, peroxidase-conjugated goat anti-mouse IgG antiserum (1:4,000; Southern Biotech) was added to the wells, and the plate was incubated for an additional period of 1 h. Peroxidase activity was revealed using hydrogen peroxide and tetramethylbenzidine (TMB). The reaction was stopped with H_2_SO_4_ (2.5 N), and the optical density (OD) at 450 nm was determined with a spectrophotometer using SOFTmax PRO 4.0 software (Life Sciences Edition; Molecular Devices Corporation, Sunnyvale, CA).

### Microneutralization *in vitro* and *in vivo*

For the microneutralization assay, the serum samples were initially diluted 1:10 and then serially diluted in 2-fold steps. The dilutions were then mixed at a 1:1 volume ratio with approximately 150 PFU of ZIKV, and the samples were incubated for 30 min at 37°C. They were then incubated with Vero cells at 60–70% confluence in 24-well culture plates for 1 h at 37°C and 5% CO_2_. Next, each well was filled with 1 mL of high-glucose DMEM containing 1% FBS, 1% 100 μg/mL penicillin, 100 μg/mL streptomycin mixed solution (LGC Biotecnologia, Cotia, SP) and 1.5% carboxymethylcellulose (CMC; Sigma-Aldrich Co, Missouri, USA). The plates were incubated at 37°C and 5% CO_2_ for 4 days. The cells were fixed by adding 1 mL of 4% formaldehyde for 30 min. Each plate was washed and stained with a crystal violet solution (1% crystal violet, 20% ethanol). The number of plaques in each well was counted to determine the neutralizing effect of the serum on the ZIKV.

For *in vivo* microneutralization, sera at a 1:4 dilution ratio were preincubated with 10^4^ ZIKV at 25°C for 30 min. Next, the AG129 mice were challenged by an intraperitoneal route with a total volume of 300 μL.

### Evaluation of protection from clinical signs and cerebral atrophy induced by ZIKV replication in the brains of neonatal mice by breastfeeding

Persistent weight loss has been associated with the severity of the ZIKV infection in mice. Therefore, on postnatal day 3 (P3), the Swiss mice were subcutaneously infected with 10^6^ PFU of ZIKV. Then, the virus-exposed pups were weighed and observed until 30 days post infection (dpi) and compared to the uninfected control group to assess clinical signs of the disease and the mortality profile. After 35 days of infection, the animals were euthanized, and their brains were removed and weighed to assess the tissue masses.

### Statistical analysis

Statistical analysis was performed using GraphPad Prism 6.01 (GraphPad). The data are reported as the means ± SEM. Tests used: log-rank (Mantel-Cox), one-way ANOVA with Tukey posttest, two-way ANOVA, and Mann-Whitney test, unpaired t-test.

## Supporting information

S1 Fig Lethal dose of ZIKV infection in A129 mice by intravenous injection. Four- to five-week-old mice were infected by the intravenous route with different concentrations of ZIKV (10^6^, 10^5^, 10^4^, or 10^3^ PFU). Mice were examined daily for survival for 15 days.
(TIF)

S2 Fig YFV vaccine promotes protection against viral propagation in cerebral tissue 35 days after immunization. **(A)** qRT-PCR of SV129 brains infected 35 days after vaccination. N = 5 statistical analysis: Student’s t-test. **p<0.01.
(TIF)

S3 Fig YFV immunization is dependent on interferon-gamma (IFN-γ). AG129 mice are highly susceptible to YFV, and they did not survive immunization. Survival after vaccination with 10^4^ and 10^2^ ZIKV. N = 11. ***p<0.0001.
(TIF)
