# Supplementary figures and images for: Yellow fever vaccine protects mice against Zika virus infection
Source: PLoS Negl Trop Dis. 2021 Nov 4;15(11):e0009907. doi: 10.1371/journal.pntd.0009907 (PMC8594798; doi:10.1371/journal.pntd.0009907)

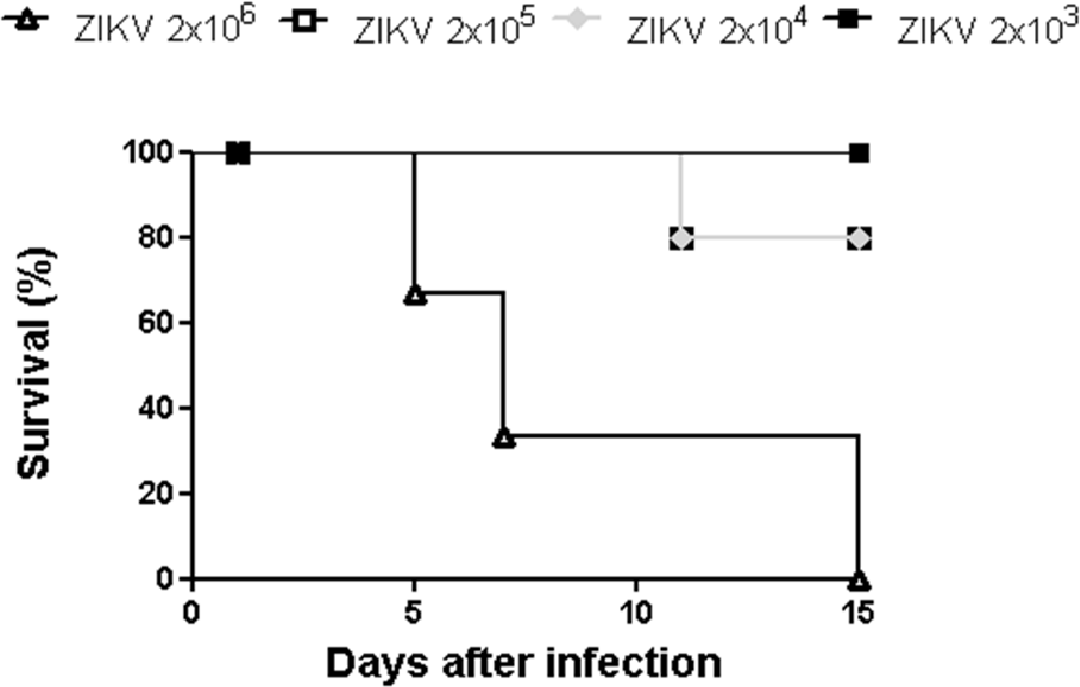

Supplement: S1 Fig — Four- to five-week-old mice were infected by the intravenous route with different concentrations of ZIKV (106, 105, 104, or 103 PFU). Mice were examined daily for survival for 15 days. (TIF) [file pntd.0009907.s001.tif]

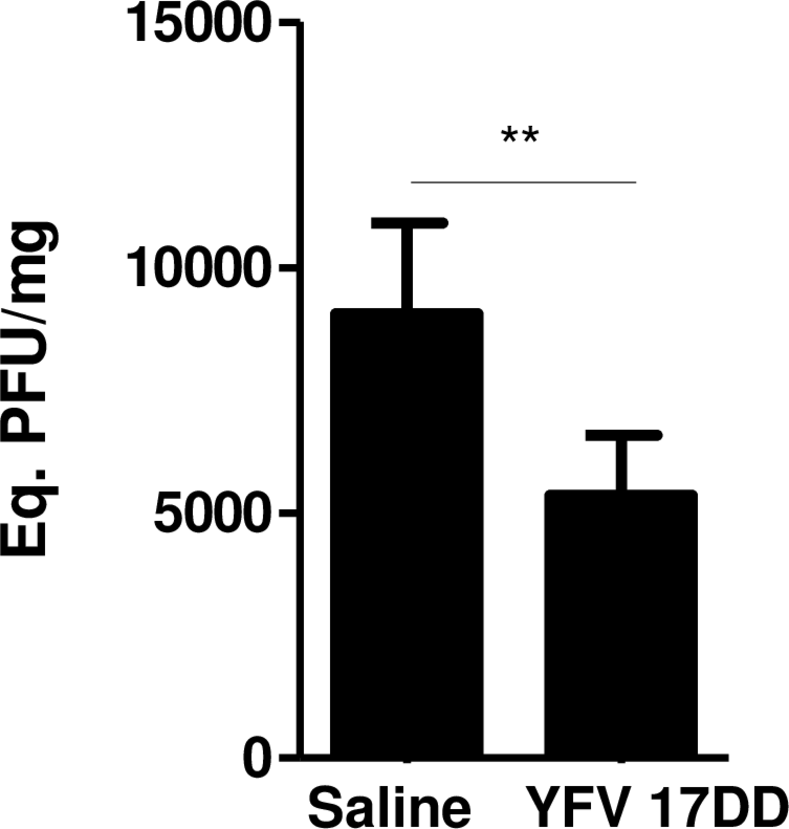

Supplement: S2 Fig — (A) qRT-PCR of SV129 brains infected 35 days after vaccination. N = 5 statistical analysis: Student’s t-test. **p<0.01. (TIF) [file pntd.0009907.s002.tif]

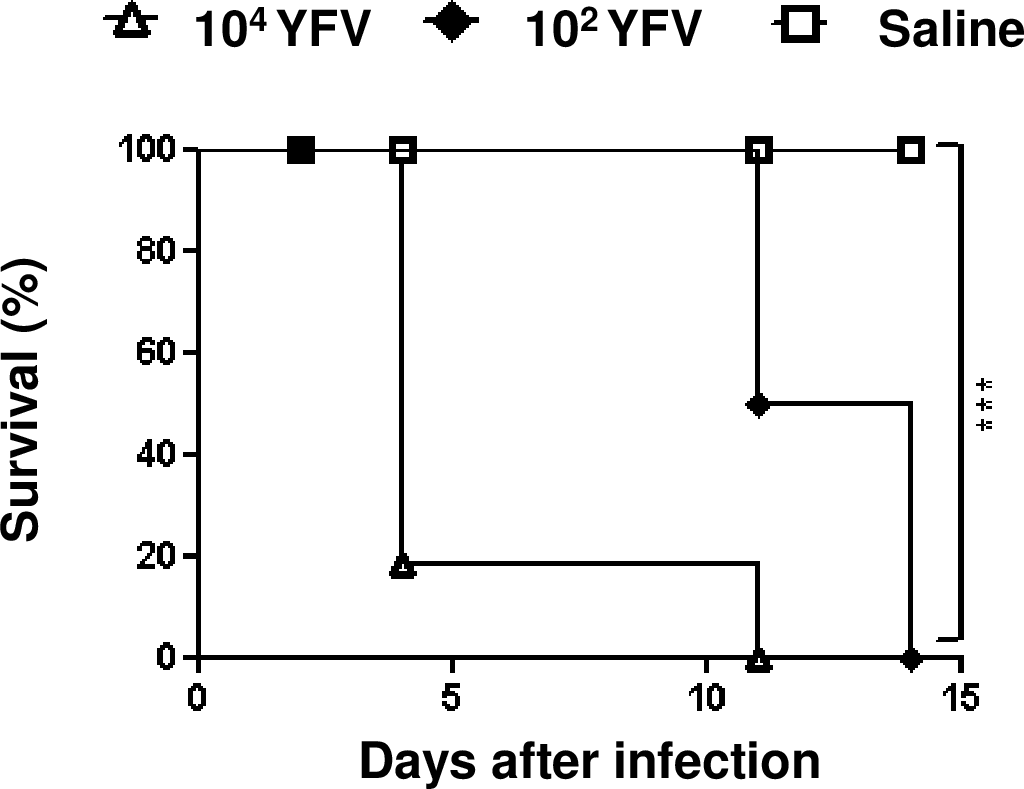

Supplement: S3 Fig — AG129 mice are highly susceptible to YFV, and they did not survive immunization. Survival after vaccination with 104 and 102 ZIKV. N = 11. ***p<0.0001. (TIF) [file pntd.0009907.s003.tif]
